# Larval Connectivity in an Effective Network of Marine Protected Areas

**DOI:** 10.1371/journal.pone.0015715

**Published:** 2010-12-21

**Authors:** Mark R. Christie, Brian N. Tissot, Mark A. Albins, James P. Beets, Yanli Jia, Delisse M. Ortiz, Stephen E. Thompson, Mark A. Hixon

**Affiliations:** 1 Department of Zoology, Oregon State University, Corvallis, Oregon, United States of America; 2 School of Earth and Environmental Science, Washington State University, Vancouver, Washington, United States of America; 3 Department of Marine Science, University of Hawaii at Hilo, Hilo, Hawaii, United States of America; 4 International Pacific Research Center, University of Hawaii, Honolulu, Hawaii, United States of America; 5 National Marine Fisheries Service, Highly Migratory Species Management Division, Silver Spring, Maryland, United States of America; 6 Marine Environmental Research, Kailua-Kona, Hawaii, United States of America; University of Canterbury, New Zealand

## Abstract

Acceptance of marine protected areas (MPAs) as fishery and conservation tools has been hampered by lack of direct evidence that MPAs successfully seed unprotected areas with larvae of targeted species. For the first time, we present direct evidence of large-scale population connectivity within an existing and effective network of MPAs. A new parentage analysis identified four parent-offspring pairs from a large, exploited population of the coral-reef fish *Zebrasoma flavescens* in Hawai'i, revealing larval dispersal distances ranging from 15 to 184 km. In two cases, successful dispersal was from an MPA to unprotected sites. Given high adult abundances, the documentation of any parent-offspring pairs demonstrates that ecologically-relevant larval connectivity between reefs is substantial. All offspring settled at sites to the north of where they were spawned. Satellite altimetry and oceanographic models from relevant time periods indicated a cyclonic eddy that created prevailing northward currents between sites where parents and offspring were found. These findings empirically demonstrate the effectiveness of MPAs as useful conservation and management tools and further highlight the importance of coupling oceanographic, genetic, and ecological data to predict, validate and quantify larval connectivity among marine populations.

## Introduction

The utility of marine protected areas (MPAs) as management and conservation tools for replenishing populations outside MPA borders depends on two processes: spillover of mobile juveniles and adults into adjacent unprotected habitat, and seeding of unprotected sites with larvae spawned within MPAs [1]. While there is mounting evidence for localized spillover [2], [3], there have been no empirically documented cases of MPAs seeding unprotected sites, which has impeded acceptance of this management tool [4]. Seeding is a form of population connectivity, which, in marine metapopulations, is characterized by the dispersal of planktonic larvae among local populations [5]. Recent empirical efforts to track larval dispersal have demonstrated localized self-recruitment [6]–[10], but have not documented larvae seeding distant or commercially fished sites.

The rarity of data demonstrating larval seeding is due to the challenges associated with documenting dispersal events in marine populations. Determining patterns of larval dispersal is especially challenging due to the minuscule sizes of larvae and the vast ocean environment through which they travel [11]. Additionally, most marine populations are characterized by high rates of genetic exchange [12] between chemically homogenous environments [13], which severely constrain available methods for determining ecologically-relevant patterns of dispersal. Therefore, we applied a new genetic parentage method [14] to directly determine how far and to what extent the larvae of an abundant coral-reef fish disperse from their natal populations.

The use of genetic parentage analyses to directly document dispersal presents a largely unexplored, yet promising alternative to traditional approaches. Until recently, parentage analyses have been methodologically constrained to environments where fishes with short pelagic larval durations occupy locations where all or most of the adults can be sampled [8], [15]. Here, we show that difficulties associated with applying parentage methods to large natural populations have been overcome with a new Bayesian approach that fully accounts for large numbers of pair-wise comparisons and unknown probabilities of finding true parent-offspring pairs [14]. This new method is well suited for a broad range of systems where only a small proportion of candidate parents can be sampled (including the majority of marine species).

**Figure 1:**
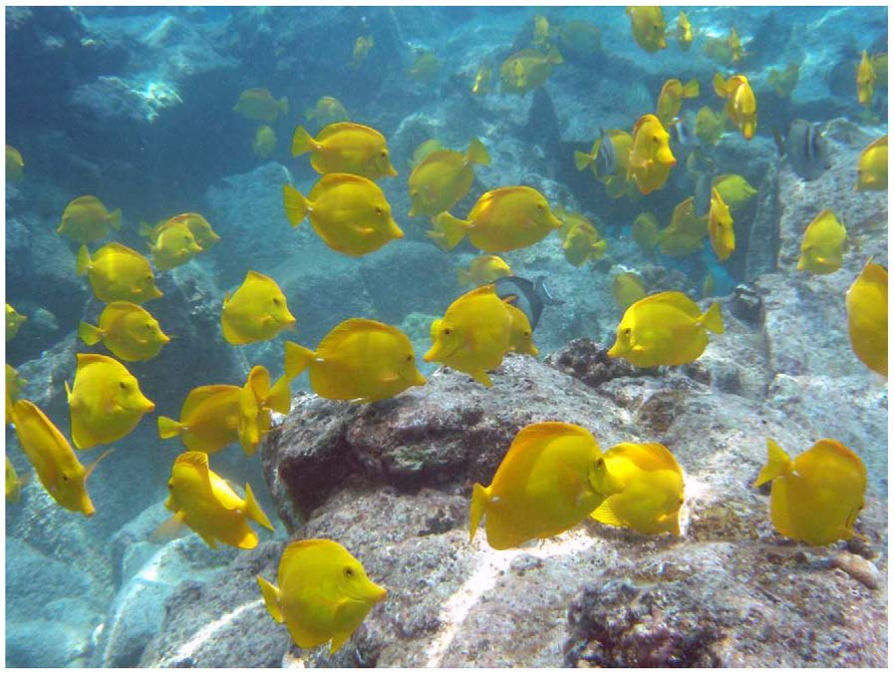
Adult yellow tang (*Zebrasoma flavescens*) photographed off the western (Kohala-Kona) coast of the Island of Hawai'i, where they occur at high densities. Approximately half a million juvenile yellow tang (representing more than 1 million U.S. dollars) are collected from the island by the aquarium industry each year. Photo: W.J. Walsh.

On coral reefs of the Island of Hawai'i, yellow tang (*Zebrasoma flavescens*) serve an important ecological role as abundant herbivores (Fig. 1) [16]. Juvenile yellow tang are also of substantial economic importance because they comprise 80% by number and 70% by value of all fish collected by the aquarium trade on the Island of Hawai'i [3]. To sustain the aquarium fishery, a network of 9 MPAs was established along the Kohala-Kona coast in 1999, resulting in the prohibition of commercial aquarium collection along 35% of the 150 km coastline of West Hawai'i [3]. After several years of protection, evidence for spillover of adults was documented near the boundaries of these MPAs [3]. This finding demonstrates that the West Hawai'i MPA network is effective in one regard, but cannot fully explain documented increases in catch of the targeted, sedentary juveniles and increased recruitment to MPAs [17]. These observations highlight the need to identify whether these MPAs successfully seed fished sites with larvae.

To address this question, we employed new genetic parentage methods to directly track successful dispersal events. We coupled these results with oceanographic analyses to determine the extent to which these events are predictable and to search for deviations from a null model of passive dispersal. Lastly, we present our parentage results within the context of *in situ* estimates of adult abundance to illustrate that the rates of connectivity between sites must be substantial. These multidisciplinary approaches are essential for informed conservation and management decisions, especially regarding the design and evaluation of marine reserves.

## Materials and Methods

### Sampling

**Figure 2:**
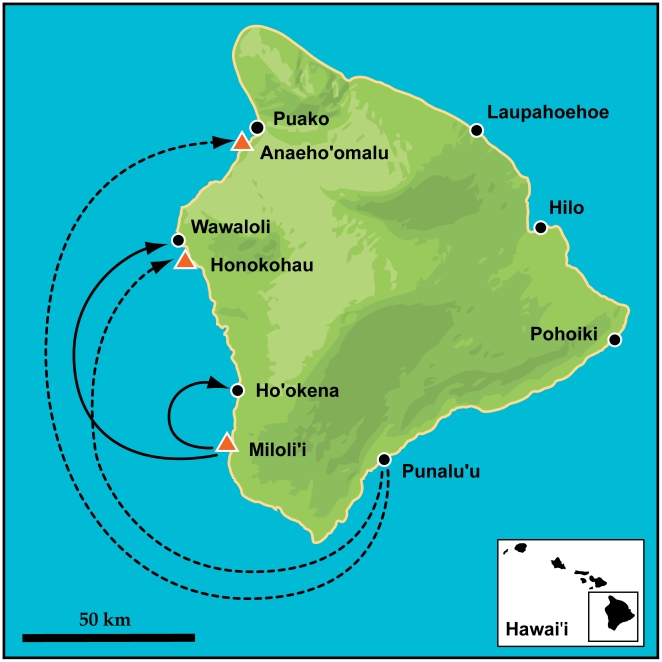
Patterns of larval connectivity in yellow tang off the Island of Hawai'i as determined by direct detection of four different parent-offspring pairs. Sample reefs are indicated by triangles and circles, where triangles represent marine protected areas (MPAs) and circles represent unprotected areas. The identified parents were sampled at Miloli'i and Punalu'u. Arrows point to the settlement site of the offspring. Solid lines indicate the first unequivocal evidence of an MPA seeding unprotected sites.

We collected tissue from 1,073 adults and recently settled juveniles yellow tang from 9 reefs located around the Island of Hawai'i (Fig. 2). Yellow tang were collected by divers on SCUBA and taken to the surface, where they were measured and had a sample of their dorsal fin tissue clipped for genetic analyses. Adults were collected from July through August 2006. Juveniles were collected from June through August 2006, the annual settlement season, with monthly collections at sites located on the west (Kohala-Kona) coast of the Island of Hawai'i (see Table 1 for sample sizes). Yellow tang were present at low densities along the entire northeast coastline and we were only able to sample one individual at Laupahoehoe.

**Table 1:** Sample sizes and population genetics summary.

Sample site*	Sample size	*K* †	Pa‡	H_O_ §	H_E_ §
Anaeho'omalu adults	49	9.24	6	**0.718**	0.796
Anaeho'omalu juveniles	82	9.03	3	0.753	0.779
Hilo adults	49	9.70	1	0.778	0.791
Hilo juveniles	42	9.57	1	0.779	0.798
Honokohau adults	73	9.32	2	0.763	0.776
Honokohau juveniles	109	9.21	5	0.767	0.799
Ho'okena adults	65	9.14	1	**0.737**	0.793
Ho'okena juveniles	68	9.44	4	0.787	0.806
Miloli'i adults	60	9.20	0	0.781	0.796
Miloli'i juveniles	67	9.38	1	**0.754**	0.809
Pohoiki adults	51	9.20	1	0.729	0.792
Pohoiki juveniles	18	9.47	1	0.811	0.796
Puako adults	66	9.21	1	0.791	0.794
Puako juveniles	48	9.26	0	0.758	0.795
Punalu'u adults	43	9.57	2	0.783	0.803
Punalu'u juveniles	49	9.38	1	0.789	0.798
Wawaloli adults	50	9.16	1	0.783	0.790
Wawaloli juveniles	83	9.26	2	**0.747**	0.792

*Age-size categories: juveniles <149 mm TL, adults >150 mm TL (total length).

†Average allelic richness per sample, rarefied to 18 individuals.

‡Number of private alleles.

§Observed (Ho) and expected (He) heterozygosities averaged across all loci. Populations where one locus deviated from HWE after a Bonferroni correction are indicated in bold.

### DNA extraction, amplification, and scoring

All fin-clip samples were stored at -20°C in 95% non-denatured ethanol. DNA was extracted using Chelex® (Biorad Laboratories) and proteinase K. PCR details are available elsewhere [18]. Samples were genotyped on an ABI 3100 capillary sequencer and scored with GENOTYPER software (Applied Biosystems). Data were scored, binned, and subsequently re-scored to check for errors. Distinct allele bins were created with FLEXIBIN [19]. Two observers independently scored 65% of all genotypes with a discordance rate of less than 0.1%. Study-specific error rates were calculated by re-genotyping 96 randomly chosen individuals at all 20 loci. All 1,073 samples were genotyped at 15 microsatellite loci [18]. Putative parent-offspring pairs were genotyped at 5 additional microsatellite loci. Additionally, a sample of 95 individuals was genotyped at the 5 additional loci employed for parentage analysis to calculate unbiased estimates of allele frequencies.

Loci were tested for deviations from Hardy-Weinberg equilibrium (HWE) in GENEPOP [20] with 10,000 batches and 5,000 iterations per batch. Tests for linkage disequilibrium were conducted in both GENETIX [21] (5,000 permutations) and GENEPOP (10,000 batches, 10,000 iterations per batch). Global and pair-wise *F_ST_* values were calculated with FSTAT [22]. Mantel tests for isolation by distance analysis were calculated with ISOLDE as implemented in GENEPOP [20].

### Parentage analyses

Because yellow tang do not move more than 1 km after settling to reef habitat [23], the along-shore distances between parents and offspring reveal the minimum dispersal distances of planktonic larvae. The genotypes of all adults were compared to the genotypes of all recruits to identify putative parent-offspring pairs that shared at least one allele at all loci. Putative pairs were genotyped at 5 additional microsatellite loci and were re-analyzed, from DNA extraction through scoring, at all 20 loci to minimize the possibility of laboratory errors. Simulations required for the calculation of 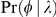, the probability of a pair being false given the frequencies of shared alleles, were conducted as recommended with 10,000 false pairs generated from over 1,000 null data sets [14]. None of the identified parent-offspring pairs had missing data, and over all samples, the amount of missing data equalled 0.6% (197 out of 33,150 scored alleles). For the calculation of false-pair probabilities only, the missing data were coded as the most common allele, which is a conservative approach. The possibility of parent-offspring pairs actually being some other first-order relative (i.e., full siblings) was eliminated by calculating the probability of simulated full sibs sharing an allele at all 20 loci (p<0.003). Simulated full sibs were created in KINGROUP [24] with the observed yellow tang allele frequencies.

### Offspring aging

Estimating the spawning and settlement dates of the offspring allowed us to examine oceanographic conditions from relevant time periods. To estimate the dates that the documented offspring were spawned, we calculated the age of the offspring on their collection date using a species-specific linear growth equation 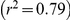. This equation was obtained by comparing the total length of 56 yellow tang recruits collected from the Island of Hawai'i (size range 30 to 47 mm) to their nearest age (in days) as determined by otolith growth rings (David J. Shafer, University of Hawai'i, *in preparation*). The total lengths of the four identified offspring ranged from 34 to 44 mm, well within the range of the available data. We subtracted the ages of the offspring from the collection date to determine the approximate date of spawning. To calculate the approximate settlement date we added the mean pelagic larval duration of yellow tang, 54 days [23], to the spawning date. Additionally, we calculated that the four offspring had lived on the reef for an average of 28 days before being sampled.

### Oceanography

We averaged measurements of sea surface height and geostrophic velocity from satellite data over the larval dispersal period of the documented offspring [25]. To construct a null model of passive larval dispersal near the Island of Hawai'i, we employed an ocean circulation model (the Hybrid Coordinate Ocean Model or HYCOM [26]) to simulate ocean flows and track virtual larvae (“drifters”) during the pelagic larval duration specific to each identified yellow tang offspring. Virtual drifters were released as close to the location of a natal site as possible, but sufficiently far from land to prevent the drifters immediately returning to shore. Initially 961 (31×31) particles were evenly distributed over rectangular patches of 0.03 degrees in width and length located at each site. All drifters were released on estimated spawning dates at depths of 1.5 and 30 meters below the sea surface (i.e., two depths simulated per site). Particle positions were sampled periodically until completion of the 54-day pelagic larval duration. The drifters were permitted to take steps in a random manner to simulate the effects of sub-grid scale processes that were not resolved by the model. The size of these steps equates to a diffusion coefficient of 10 m^2^s^−1^.

### Abundance estimates

We first employed site-specific density estimates of yellow tang to calculate population sizes from reefs with documented parents (see Text S1 in File S1 for details). We also calculated estimates of the total population size for the entire island by using summed habitat stratified density estimates within nine MPAs (see methods in [27]). Density estimates for non-MPA areas were based on mean values for areas open to fishing along the Kona-Kohala coast [3]. East Hawaii estimates were based on average density estimates for four sites. Total population size was calculated using the sum of density estimates for each area multiplied by the total reef area. Using these abundance data, we estimated the rates of connectivity between sites where parents and offspring were sampled. Because there is a large degree of uncertainty in these estimates, we present and discuss these calculations only in the File S1.

## Results

We identified four parent-offspring pairs (Fig. 2), which is remarkable given the approximately 54-day pelagic larval duration and the large number of yellow tang around the Island of Hawai'i. The study specific error-rate of 0.008 allowed for up to 2 loci to mismatch [14], though all documented parent-offspring pairs mismatched at either 0 or 1 locus (2 pairs each). All offspring were assigned to a different parent and were unrelated (no alleles in common). The probability of parent-offspring pairs sharing alleles by chance was low, ranging from 0.001 to 0.027 (Table 2). Importantly, this false pair probability represents the probability of a parent-offspring pair being false after accounting for the frequencies of shared alleles and all pair-wise adult-juvenile comparisons.

**Table 2:** Locations of parent-offspring pairs and corresponding probabilities of sharing alleles by chance (false pair probabilities), along-shore dispersal distances.

Sample reef		False pair probability	Dispersal distance
Parent	Offspring	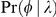	(km)
Miloli'i	Ho'okena	0.0038	15.4
Miloli'i	Wawaloli	0.0013	64.9
Punalu'u	Honokohau	0.0272	140.1
Punalu'u	Anaeho'omalu	0.0109	184.2

Average observed heterozygosity was 0.764 with an average of 12 alleles per locus (range: 4 to 28). No loci were out of HWE at more than one sample site, and 14 out of 18 sample sites did not have any loci out of HWE (Table 1). There was no evidence for linkage disequilibrium among all pairs of loci. We could not reject a null hypothesis of genetic homogeneity (panmixia) among all sampled reefs (F_ST_: 99% confidence interval  = **−**0.001–0.001; pooled samples in Hardy-Weinberg Equilibrium). Furthermore, we did not find any evidence for isolation-by-distance using nearest along-shore distances (p>0.54; Figure S1). Adjusting for marker polymorphism by calculating Hedrick's G_ST_ [28] did not change any of the above conclusions (e.g., more than half of the pair-wise F_ST_ values were negative).

**Figure 3:**
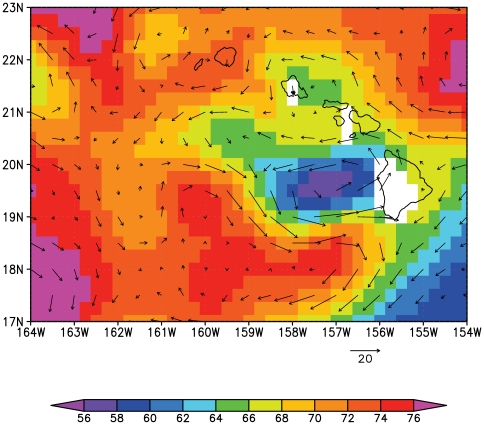
Sea surface height (cm, colors) as observed by satellites and the geostrophic velocity (cm/s, vectors) derived from satellite altimetry and averaged over the larval dispersal period of the four documented offspring shown in **Figure 2**. The cyclonic eddy is indicated by low sea surface heights and anti-clockwise rotation west of the Island of Hawai'i.

**Figure 4:**
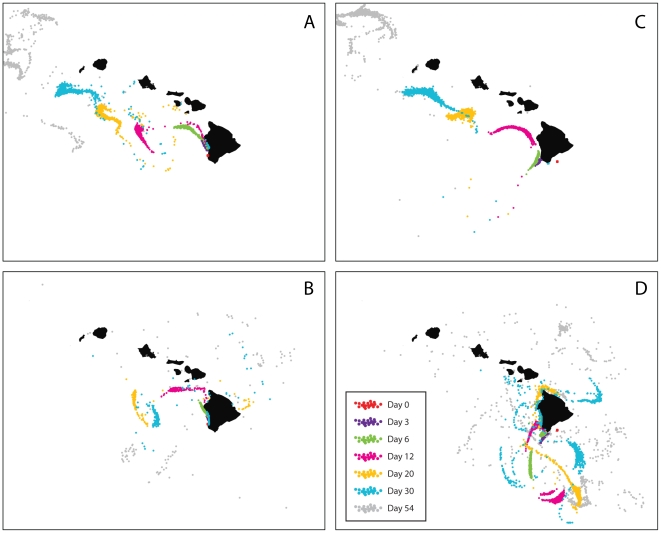
Dispersal of 1000 passive virtual drifters for 54 days – the pelagic larval duration of yellow tang – released from the two reefs where parents were identified. Shown are passive dispersal of drifters released from Miloli'i on the date of spawning of a documented offspring (26 April 2006) at (**A**) sea surface level and (**B**) 30 meters below the sea surface, as well as passive dispersal of drifters released from Punalu'u at the date of spawning of another documented offspring (24 April 2006) at (**C**) sea surface level and (**D**) 30 meters below the sea surface. For drifters released at sea level, initial post-spawning dispersal was northward, followed by subsequent dispersal to the northwest. Drifters released at 30 meters below sea surface remained closer to the Island of Hawai'i and clustered near sites where offspring were identified. Note that yellow tang release floating gametes near the ocean surface and that the behavior of older larvae is presently unknown.

All identified offspring were found between 15 and 184 kilometers to the north of their parents, suggesting that ocean currents played a substantial role in larval dispersal. Satellite altimetry data revealed a large cyclonic meso-scale eddy that created northward currents along the coast of the island (Fig. 3). The eddy persisted from early April to mid-June 2006 before moving slowly westward. Such eddies occur frequently in this region in response to the prevailing northeasterly trade winds [29]. The eddies are surface-intensified but their influence can extend to over 200 m in depth. The observed cyclonic eddy was reproduced in the HYCOM model simulation. Virtual drifters released at the ocean surface, initially moved northward along coastline, but subsequently drifted to the northwest (Fig. 4a, c). Drifters released at 30 m below sea level, were retained near the Island of Hawai'i in greater numbers (Fig. 4b, d). The behavior of yellow tang larvae is presently unknown, yet it seems likely that older larvae may take active measures (e.g., change depth) to avoid being swept far offshore [30]. Surgeonfish (tang) eggs are buoyant and pelagic until hatching (∼1–2 days) and young larvae are likely to be fairly poor swimmers [31]. Late-stage surgeonfish larvae, however, have been observed to occur in water as deep as 100 meters and to be strong swimmers [32]–[36]. Thus, it is likely that yellow tang larvae are initially passive and become progressively stronger swimmers with age.

We conservatively estimated the total yellow tang population size from the Island of Hawai'i to be 4.2±2.2 million individuals. Using the adult abundance data estimated from each site (Table S1 in File S1) we estimate that we sampled an average of only 0.06% (range: 0.02–0.2%) of the adults at reefs where parents were identified. Thus, the large population size coupled with the small proportion of individuals sampled strongly suggests that (1) yellow tang have unequal reproductive success (i.e., if yellow tang had equal reproductive success, then we would be very unlikely to sample any parent-offspring pairs), and (2) the rates of larval connectivity between sites must be ecologically significant.

## Discussion

The new parentage approach demonstrated here provides a means of directly documenting larval dispersal at higher resolution than conventional indirect means. We detected dispersal distances up to 184 km, which is a substantially greater distance than previously detected using other direct methods [12]. Because we could measure only the nearest along-shore distances, the actual distances travelled by larvae may be considerably greater, particularly if they were entrained in the near-shore eddy. Importantly, our results do not support a pattern of high local larval retention, which has been indicated by other parentage studies [6]–[10]. One possible explanation is that previous studies focused predominantly on anemonefishes and damselfishes (Family: Pomacentridae). In our study, the small proportion of sampled adults coupled with the observation that the tracked larvae survived to become established juveniles, demonstrates high rates of ecologically meaningful population connectivity among these reefs around the Island of Hawai'i. These results provide new insight on the ecologically important process of dispersal, namely that (1) adult yellow tang populations are highly connected by larval dispersal, and (2) along-shore distances do not appear to limit yellow tang dispersal at the island-wide scale (see [37] for an among-island study).

Given what is known about surgeonfish larval ecology, it is likely that young yellow tang larvae were initially transported passively by ocean currents [31] and may even have been transported and entrained within the observed ocean eddy [38]. As the larvae grew larger, they were likely able to change depth and eventually become relatively strong swimmers [32]–[36]. We speculate that active behavioural mechanisms prevented the larvae from becoming permanently entrained in the observed eddy and allowed them to successfully settle to suitable coral-reef habitat [38]. Additionally, our results indicate that dispersal trajectories may be predictable with oceanographic analyses (see also [39]–[41]). Increases in the accuracy and precision of oceanographic methods will come from accurate near-shore oceanographic modelling, species-specific knowledge of larval behavior, and further empirical validation. The continued refinement and integration of genetic and oceanographic methods will lead to appropriate design decisions (e.g., size, spacing, location) that will allow for marine reserves to better meet their goals [4], [42].

Lack of unequivocal evidence for the hypothesized seeding effect has long impeded acceptance of MPAs as useful tools for marine fisheries management and conservation. Our observations of larval connectivity provide the first direct evidence of marine protected areas (MPAs) successfully seeding unprotected areas with larval fish. In fact, the ‘unprotected’ site with identified parents (Punalu'u) is functionally similar to an MPA because: (1) almost all collection of yellow tang occurs on the Kohala-Kona coast, and (2) this site is logistically difficult to access for collection purposes due to high wave exposure. Thus, both reefs where parents were identified were not substantially fished and they clearly seeded both MPAs and reefs that are open to fishing.

In addition to demonstrating the seeding effect of MPAs, documenting connectivity among marine populations has an important social and economic role. The identification of connectivity between distant reef fish populations on the Island of Hawai'i demonstrates that human coastal communities are also linked: management in one part of the ocean affects people who use another part of the ocean. Understanding connections at all levels is the foundation for truly effective ecosystem-based management [43].

## Supporting Information

Figure S1 **Test of isolation‐by‐distance in yellow tang collected from the Island of Hawai'i.** Adult and juvenile samples were treated as separate populations. Mantel tests were run in GENEPOP with both normal and log‐transformed distances and with F_ST_ and F_ST_/(1‐F_ST_). Tests could not reject the null hypothesis of no isolation‐by distance. At the within‐island scale, there is no increase in genetic differentiation between populations as the distance between populations increases. The dashed line represents a best‐fit linear model.
(DOC)

File S1 **Description of the methods employed to calculate the abundance of yellow tang and estimates of connectivity between sites where parents and offspring were identified (Text S1)**. We also include a table of adult abundance estimates (Table S1) and estimated connectivity between sites (Table S2).
(DOC)
