# Supplementary material for: Larval Connectivity in an Effective Network of Marine Protected Areas
Source: PLoS One. 2010 Dec 21;5(12):e15715. doi: 10.1371/journal.pone.0015715 (PMC3006342; doi:10.1371/journal.pone.0015715)

**Supplemental Figure 1:** Test of isolation-by-distance in yellow tang collected from the Island of Hawaii. Adult and juvenile samples were treated as separate populations. Mantel tests were run in GENEPOP with both normal and log-transformed distances and with F_ST_ and F_ST_/(1-F_ST_). Tests could not reject the null hypothesis of no isolation-by distance. At the within-island scale, there is no increase in genetic differentiation between populations as the distance between populations increases. The dashed line represents a best-fit linear model.


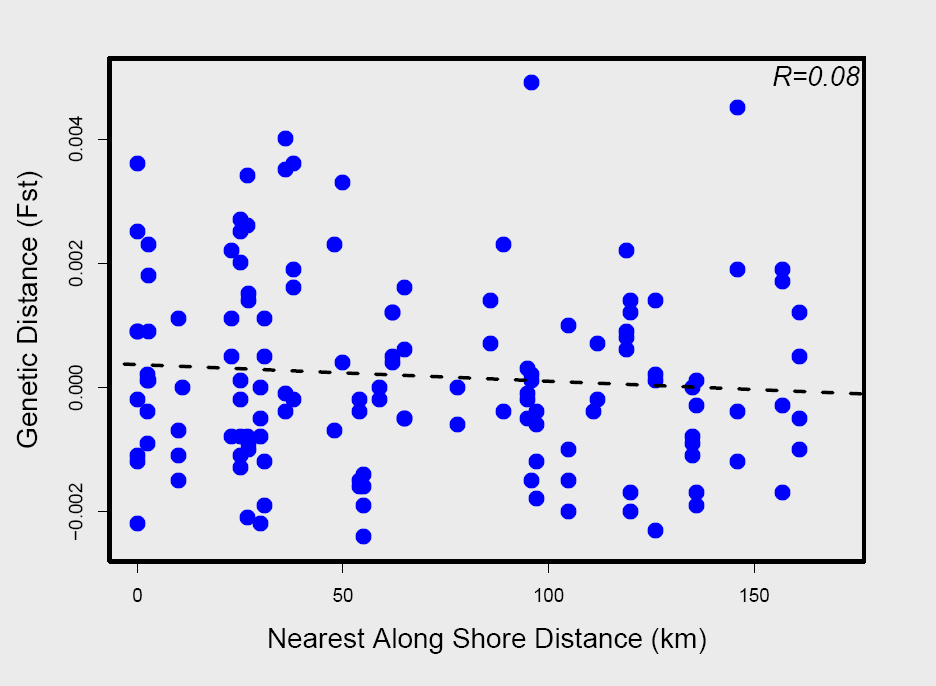

Supplement: Figure S1 — Test of isolation‐by‐distance in yellow tang collected from the Island of Hawai'i. Adult and juvenile samples were treated as separate populations. Mantel tests were run in GENEPOP with both normal and log‐transformed distances and with FST and FST/(1‐FST). Tests could not reject the null hypothesis of no isolation‐by distance. At the within‐island scale, there is no increase in genetic differentiation between populations as the distance between populations increases. The dashed line represents a best‐fit linear model. (DOC) [file pone.0015715.s001.doc]
