# Supplementary material for: Larval Connectivity in an Effective Network of Marine Protected Areas
Source: PLoS One. 2010 Dec 21;5(12):e15715. doi: 10.1371/journal.pone.0015715 (PMC3006342; doi:10.1371/journal.pone.0015715)
Supplement: File S1 — Description of the methods employed to calculate the abundance of yellow tang and estimates of connectivity between sites where parents and offspring were identified (Text S1). We also include a table of adult abundance estimates (Table S1) and estimated connectivity between sites (Table S2). (DOC) [file pone.0015715.s002.doc]

Supporting File S1

*Text S1: Abundance and connectivity estimates*

Using a habitat-stratified sampling approach, yellow tang densities were estimated between May and July using a total of 83 circular plot counts (78 m^2^ plot area), ranging from 17 to 25 plots per site. Survey locations within specific habitat types were determined from a map of reef habitats developed for our study sites (Ortiz and Tissot 2008). The number of samples among habitat types and sites was based on the proportional area of the various habitats at each site. Fish sampling followed the methodology previously described in Ortiz and Tissot 2008. Adult abundances were calculated by multiplying the mean density (no. m^-2^) of adults by the total reef area (m^2^) for each habitat, and then summing the habitat-specific estimates to produce total adult abundance for each reef (Table S1).

We next calculated *P*, which is the proportion of offspring expected to be in our sample of recruits if we had sampled 100% of the adults from the site where a parent was found:

(1)

where *N_O_* is the total number of offspring identified at a site via parentage analysis, and *N_R_* is the sample size of recruits from which an offspring was identified. Thus *N_O_ /N_R_*  equals the proportion of identified offspring from a given site in a sample of recruits. This quantity is divided by the proportion of adults sampled at a site with an identified parent (*M/N_A_*), where *M* equals the number of adults sampled at a site where the parent was identified and *N_A_* is the total number of adults at that site (determined with density estimates). Assuming that the sample of recruits is representative, *P* equals the proportion of recruits at site *x* that came from site *y*. Confidence intervals were calculated by employing the 95% confidence intervals from our adult abundance estimates (Table S2).

References:

**Ortiz DM, Tissot BN (2008) Ontogenetic patterns of habitat use by reef-fish in a Marine Protected Area network: a multi-scaled remote sensing and in situ approach. *Marine Ecological Progress Series* 365:217-232.**

*Table S1:* Estimates of adult abundance for each site where a parent or offspring was found ± 95% confidence interval. Notice the high adult abundances and the small proportion of adults sampled at each site.

| Sample site | Adult abundance | 95% ± | Site area (m^2^) | Percent sampled |
| --- | --- | --- | --- | --- |
| Anaeho'omalu | 159,101 | 67,517 | 5,248,700 | 0.031 |
| Wawaloli | 95,000 | 52,000 | 1,100,000 | 0.053 |
| Honokohau | 72,521 | 36,160 | 1,124,500 | 0.101 |
| Ho'okena | 20,087 | 12,425 | 2,051,000 | 0.323 |
| Miloli'i | 82,651 | 57,742 | 3,361,000 | 0.073 |
| Punalu'u | 114,594 | 70,774 | 1,327,856 | 0.038 |

*Table S2:* Estimated rates of larval connectivity from parent to offspring reefs (with 95% confidence intervals). These estimates equal the proportion of recruits at a site where an offspring was identified that came from a site where a parent was identified (e.g., 20% of recruits at Milolii came from Hookena). Note that there are many assumptions that go into this estimate including: (1) the number and occurrence of parent-offspring pairs are representative of the larger population, (2) sampling was completely random (including with respect to the reproductive success of individual fish). The main point is that during our sampling period, which coincided with northward currents, there was a substantial amount of larval connectivity between sites.

| Sample reef | | Connectivity |
| --- | --- | --- |
| Parent | Offspring | % (with 95% CI) |
| Miloli'i | Ho'okena | 20.3 (6.1-34.4) |
| Miloli'i | Wawaloli | 16.6 (5.0-28.8) |
| Punalu'u | Honokohau | 24.5 (9.3-39.5) |
| Punalu'u | Anaeho'omalu | 46.8 (17.9-75.6) |
